# Study on Micro-Crack Induced Precision Severing of Quartz Glass Chips

**DOI:** 10.3390/mi9050224

**Published:** 2018-05-08

**Authors:** Long Zhang, Jin Xie, Aodian Guo

**Affiliations:** School of Mechanical and Automotive Engineering, South China University of Technology, Guangzhou 510640, China; zhanglong1226@126.com (L.Z.); aodianguo@163.com (A.G.)

**Keywords:** micro-crack propagation, severing force, quartz glass, micro-grinding

## Abstract

It is difficult to cut hard and brittle quartz glass chips. Hence, a method involving micro-crack-induced severing along a non-crack microgroove-apex by controlling the loading rate is proposed. The objective is to realize the rapid and precision severing of the hardest quartz glass in chip materials. Firstly, micro-grinding was employed to machine smooth microgrooves of 398–565 μm in depth; then the severing force was modelled by the microgroove shape and size; finally, the severing performance of a 4-mm thick substrate was investigated experimentally. It is shown that the crack propagation occurred at the same time from the microgroove-apex and the loading point during 0.5 ms in micro-crack-induced severing. The severing efficiency is dominated by the severing time rather than the crack propagation time. When the loading rate is less than 20–60 mm/min, the dynamic severing is transferred to static severing. With increasing microgroove-apex radius, the severing force decreases to the critical severing force of about 160–180 N in the static severing, but it increases to the critical severing force in the dynamic severing. The static severing force and time are about two times and about nine times larger than the dynamic ones, respectively, but the static severing form error of 16.3 μm/mm and surface roughness of 19.7 nm are less. It is confirmed that the ideal static severing forces are identical to the experimental results. As a result, the static severing is controllable for the accurate and smooth separation of quartz glass chips in 4 s and less.

## 1. Introduction

Rolling scribing with a tungsten carbide or polycrystalline diamond (PCD) wheel is widely used to separate silicate glass substrates without any coolant and material removal. In order to improve the life of tool micro-tips, a chemical vapor deposition (CVD) diamond roller was developed for scribing instead of PCD and carbide alloy rollers [1]. Lateral and radial cracks were, however, produced on severing surfaces due to the mechanical force of mechanical wheel rolling [2,3]. Generally, a follow-on smooth profile grinding and polishing were needed along with inefficiency and pollution. Recently, researchers have focused on the crack generation on severing surfaces. From the in-process estimation of fracture surface morphology, severing surface cracks and breakages were produced during wheel scribing of a glass sheet [4]. It has been known that the median crack depth decreased with decreasing loading force, but it still reached about 90 μm with the load of 22 N in scribing alumina ceramics [1]. In scribing LCD glass, it reached about 45 μm with the load of 5.5 N [2] and about 90 μm with the cutting pressure of 0.16 MPa [3], respectively. Until now, the scribing of harder quartz glass chips has not yet been reported.

In order to improve scribing performance, vibration-assisted scribing was used to increase the median cracks for severing [5], but uneven cracks existed on microgroove-apex edges and the severing surface. It has been reported that laser beams could be used to irradiate the scribing [6,7,8,9]. Although laser beam irradiation enhanced scribing speed, edge cracks, severing form deviation and the bevel surfaces were produced [6]. Moreover, the laser irradiation made the mechanical breaking easier in scribing [7], but thermal damage accumulated at the severing edges. Because the laser scribing produced micro-cracks and burrs on the machined microgrooves, it led to the cracks and burrs on the severing surface edge [8]. Although a hybrid of laser beam, water jet coolant and pre-bending were employed to eliminate the micro-cracks [9], form deviation happened along the beam moving direction. A picosecond Ultraviolet (UV) laser was also used to induce the scribing of polyethylene terephthalate films to control the local bending flexibility [10], but it has not yet been applied to severing.

To predict severing force, Filippi first proposed the existence of linear elastic stress fields in the neighborhood of rounded-tip V-shaped notches [11]. The linear elastic stress was also used to derive two brittle fracture criteria such as mean stress (MS) and point stress (PS) criteria [12]. Moreover, these criteria were used to predict compressive notch fracture toughness [13,14]. The minimum fracture loading of a U-notches plate was introduced by means of MS and PS criteria [15]. In the case of low and high loading rates, it was found that the loading rate produced little influence on the maximum load for the V-notch on fracture [16]. However, these workpieces only concerned easy-to-cut polymeric and metallic materials. Until now, these criteria have not been applied to difficult-to-cut quartz glass due to the fabrication difficulty of microgroove. Although the fracture of ceramic-metal joint surface has been divided into static and dynamic states [17], the critical loading rate and force have not yet been studied in detail.

As for the fabrication of microgroove, laser and etching approaches have been used to fabricate the microgroove with 7.5 μm and less in depth on Si surface and ceramic cylinder [18,19], but it was irregular and rough. It would lead to cracks on the severing surface when it was used for the induced severing. Moreover, the micro-grinding with a sharpened diamond wheel micro-tip may be employed to fabricate accurate and smooth microgrooves on difficult-to-cut silicon, carbide alloy and glass surfaces [20], but it has not yet been applied to the crack propagation for precision and smooth severing of difficult-to-cut materials.

In this paper, a new micro-crack-induced severing with static loading and dynamic loading is proposed for the crack propagation along an accurate and smooth microgroove-apex. The objective is to realize rapid and precision severing of difficult-to-cut quartz glass. Firstly, the trued diamond wheel micro-tip was employed to grind the accurate and non-crack microgroove on workpiece surface; then the severing force was modelled in micro-crack induced severing by microgroove parameters and loading rate; finally, severing force, severing time, cracking propagation time, severing form errors and severing surface roughness were experimentally investigated.

## 2. Micro-Crack Induced Severing of Brittle Workpiece

**Figure 1:**
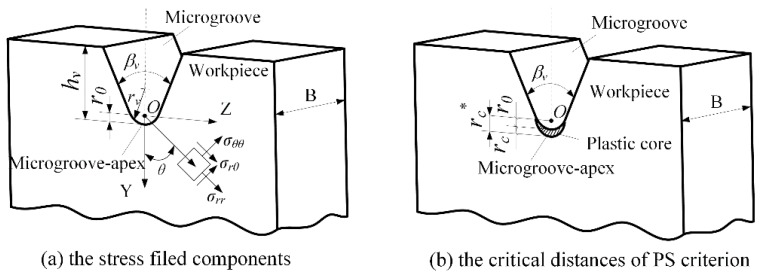
The stress field model in micro-crack induced severing along a microgroove-apex: (**a**) the stress filed components and (**b**) the critical distances of point stress (PS) criterion.

Figure 1 shows the stress field model in micro-crack induced severing along a microgroove-apex. The microgroove is parameterized by height *h_v_*, angle *β_v_* and microgroove-apex radius *r_v_*. Under mode I loading condition, *σ_θθ_*, *σ_rθ_*, and *σ_rr_* are tangential stress, shear stress and radial stress, respectively, and r_c_^*^ is critical distance (see Figure 1a) [11]. When the loading force *F* increases to the critical value called severing force *F_c_*, the tangential stress *σ_θθ_* (r_c_^*^, 0) reaches the ultimate tensile strength *σ_μ_* and the micro-cracks are produced from the microgroove-apex (see Figure 1b). It leads to the crack propagation along the microgroove-apex.

**Figure 2:**
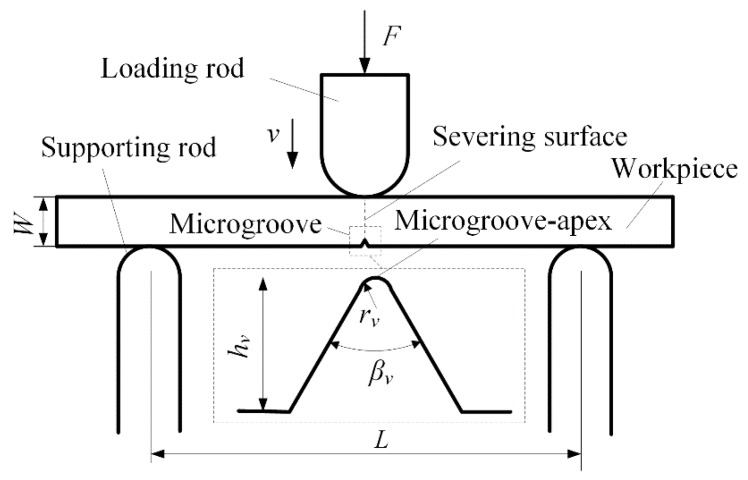
The scheme of micro-crack induced severing.

Figure 2 shows the scheme of micro-crack-induced severing. The working sizes of the workpiece substrate are given by the thickness *W* and the width *B*. The workpiece is supported by two supporting rods with an interval *L*. The arc-shaped loading rod is loaded on the upper surface of substrate. The microgroove-apex is positioned on the opposite side of substrate. The vertical loading direction aims to the microgroove-apex. The loading rod moved vertically with the loading rate *v*. When the loading force *F* reaches the severing force *F_c_*, the micro-crack occurs at the microgroove-apex. It leads to the severing for the separation of workpiece.

## 3. Modelling of Severing Force

The elastic stresses were described at the neighborhood of microgroove-apex in polar coordinate system (see Figure 1a). *r_0_* is the distance between coordinate origin *O* and microgroove-apex. The critical distances r_c_^*^ and *r_c_* are defined from the coordinate origin *O* and the microgroove-apex, respectively (see Figure 1b). The ideal severing force F_c_^*^ was described as follows [15]:

(1) $$\sigma_{\max}=\sigma_{\mathrm{nom}}K_{t}=\frac{3K_{t}F_{c}^{*}L}{2B{\left(W-h_{v}\right)}^{2}}$$

where *σ*_max_ and *σ*_nom_ are the maximum stress and the nominal stress at microgroove-apex, respectively. *K_t_* is the stress concentration factor.

At the neighborhood of microgroove-apex under pure mode I loading, the elastic stresses are described in the polar coordinate system as follows [11]:

(2) $$\left\{\begin{array}{c} \sigma_{\theta \theta }\\ \sigma_{rr}\\ \sigma_{r\theta } \end{array}\right\}=\frac{K_{Ι}^{V,r_{v}}}{\sqrt{2\pi }r^{1-\lambda_{1}}}\left[\left\{\begin{array}{c} m_{\theta \theta }\left(\theta \right)\\ m_{rr}\left(\theta \right)\\ m_{r\theta }\left(\theta \right) \end{array}\right\}+{\left(\frac{r}{r_{0}}\right)}^{\mu_{1}-\lambda_{1}}\left\{\begin{array}{c} n_{\theta \theta }\left(\theta \right)\\ n_{rr}\left(\theta \right)\\ n_{r\theta }\left(\theta \right) \end{array}\right\}\right]$$

where the *m_θθ_*(*θ*) is expressed as follows:

(3) $$\left\{\begin{array}{c} m_{\theta \theta }\left(\theta \right)\\ m_{rr}\left(\theta \right)\\ m_{r\theta }\left(\theta \right) \end{array}\right\}=\frac{1}{\left[1+\lambda_{1}+\chi_{b_{1}}\left(1-\lambda_{1}\right)\right]}\left[\left\{\begin{array}{c} \left(1+\lambda_{1}\right)\cos\left(1-\lambda_{1}\right)\theta \\ \left(3-\lambda_{1}\right)\cos\left(1-\lambda_{1}\right)\theta \\ \left(1-\lambda_{1}\right)\sin\left(1-\lambda_{1}\right)\theta \end{array}\right\}+\chi_{b_{1}}\left(1-\lambda_{1}\right)\left\{\begin{array}{c} \cos\left(1+\lambda_{1}\right)\theta \\ -\cos\left(1+\lambda_{1}\right)\theta \\ \sin\left(1+\lambda_{1}\right)\theta \end{array}\right\}\right]$$

The *n_θθ_*(*θ*) is expressed as:

(4) $$\left\{\begin{array}{c} n_{\theta \theta }\left(\theta \right)\\ n_{rr}\left(\theta \right)\\ n_{r\theta }\left(\theta \right) \end{array}\right\}=\frac{1}{4\left(q-1\right)\left[1+\lambda_{1}+\chi_{b_{1}}\left(1-\lambda_{1}\right)\right]}\left[\chi_{d_{1}}\left\{\begin{array}{c} \left(1+\mu_{1}\right)\cos\left(1-\mu_{1}\right)\theta \\ \left(3-\mu_{1}\right)\cos\left(1-\mu_{1}\right)\theta \\ \left(1-\mu_{1}\right)\sin\left(1-\mu_{1}\right)\theta \end{array}\right\}+\chi_{c_{1}}\left\{\begin{array}{c} \cos\left(1+\mu_{1}\right)\theta \\ -\cos\left(1+\mu_{1}\right)\theta \\ \sin\left(1+\mu_{1}\right)\theta \end{array}\right\}\right]$$

The *K*_I_*^V,rv^* is the mode I notch stress intensity factor (NSIF). It is described as follows:

(5) $$K_{Ι}^{V,r_{v}}=\frac{\sqrt{2\pi }r_{0}{}^{1-\lambda_{1}}\sigma_{\max}}{1+\omega_{1}}$$

where *ω*_1_ is an auxiliary parameter. They are expressed as follows:

(6) $$r_{0}=\frac{q-1}{q}r_{v}$$

(7) $$\omega_{1}=\frac{q}{4\left(q-1\right)}\left[\frac{\chi_{d_{1}}\left(1+\mu_{1}\right)+\chi_{c_{1}}}{1+\lambda_{1}+\chi_{b_{1}}\left(1-\lambda_{1}\right)}\right]$$

where *q* is a real positive coefficient ranging as:

(8) $$q=\frac{2\pi -\beta_{v}}{\pi }$$

*λ*_1_, *μ*_1_, *χ_b_*_1_, *χ_c_*_1_ and *χ_d_*_1_ are the values of auxiliary parameters for different microgroove angle [11]. Under pure mode I loading, the tangential stress *σ_θθ_* (*r*, 0) from Equation (2) can be written as follows:

(9) $$\sigma_{\theta \theta }\left(r,0\right)=\frac{K_{Ι}^{V,r_{v}}}{\sqrt{2\pi }r^{1-\lambda_{1}}}\left[1+{\left(\frac{r}{r_{0}}\right)}^{\mu_{1}-\lambda_{1}}n_{\theta \theta }\left(0\right)\right]$$

Substituting Equation (5) into Equation (9), the tangential stress can be expression as follows:

(10) $$\sigma_{\theta \theta }\left(r,0\right)=\frac{r_{0}{}^{1-\lambda_{1}}\sigma_{\max}}{r^{1-\lambda_{1}}\left(1+\omega_{1}\right)}\left[1+{\left(\frac{r}{r_{0}}\right)}^{\mu_{1}-\lambda_{1}}n_{\theta \theta }\left(0\right)\right]$$

According to PS criterion, the brittle fracture takes place when the tangential stress *σ_θθ_* (*r*, 0) reaches critical value *σ_u_* at specified critical distance [21]. Hence, r_c_^*^ (see in Figure 1b) can be expressed as follows:

(11) $$r_{c}^{*}=r_{c}+r_{0}$$

For brittle materials, the critical distance *r_c_* can be written as follows [22]:

(12) $$r_{c}=\frac{1}{8\pi }{\left(\frac{K_{IC}}{\sigma_{u}}\right)}^{2}$$

where *K_IC_* is the material attribute called fracture toughness.

According to Equations (1) and (9)–(12), the ideal severing force F_c_^*^ is deduced as follows:

(13) $$F_{c}^{*}=\frac{2\sigma_{u}B{\left(W-h_{v}\right)}^{2}\left(1+\omega_{1}\right){\left(r_{0}+r_{c}\right)}^{1-\lambda_{1}}}{3K_{t}Lr_{0}^{1-\lambda_{1}}\left(1+{\left(1+\frac{r_{c}}{r_{0}}\right)}^{\mu_{1}-\lambda_{1}}n_{\theta \theta }\left(0\right)\right)}$$

In Equation (13), the *K_t_* is achieved in the case of the U-notch with *β_v_* = 0 [15], but it was calculated by the fitting of experimental data in this study. This is because a microgroove with *β_v_* > 0 was employed in micro-crack-induced severing.

## 4. Micro-Grinding of Microgroove on Workpiece Surface

**Figure 3:**
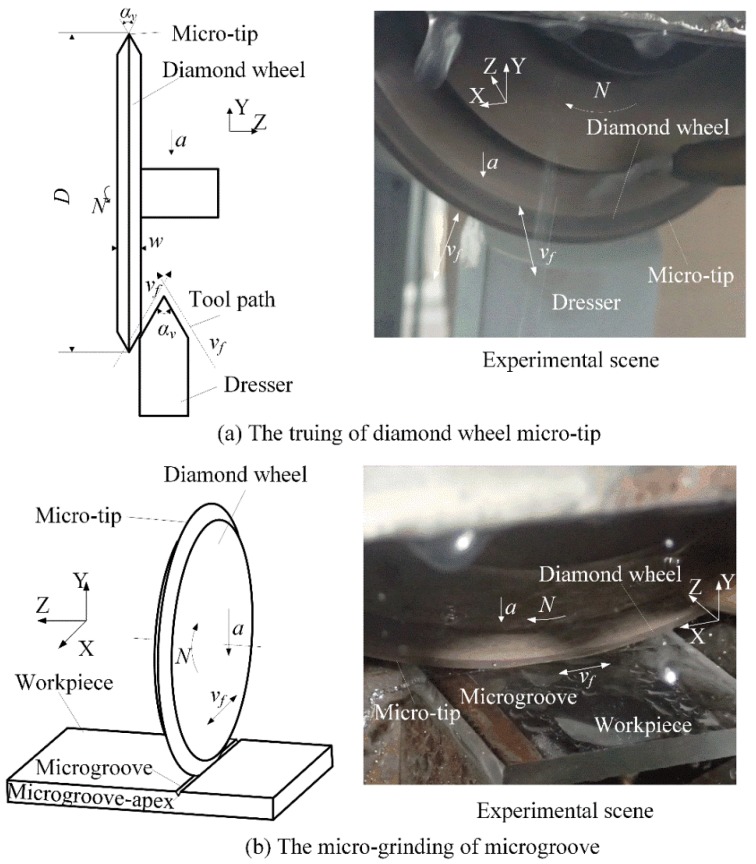
Micro-grinding of microgroove on quartz glass: (**a**) the truing of diamond wheel micro-tip and (**b**) the micro-grinding of microgroove.

Figure 3 shows the micro-grinding of microgrooves on a workpiece surface with a diamond wheel micro-tip. It was difficult to dress the wheel micro-tip due to its high hardness. Before micro-grinding, the truing of the #600 metal-bond diamond wheel micro-tip (Changxing technology Co. LTD., Shenzhen, China) was performed by the Numerical Control (NC) mutual wearing between the diamond wheel and the #800 Green Silicon Carbide (GC) dresser using Computer Numerical Control (CNC) grinder (Fuyu Machine Tool co. LTD., Zhang Hua, Taiwan) (see Figure 2a) [20]. Finally, the diamond wheel micro-tip angle *α_v_* was identical to the angle of the NC tool paths. The truing conditions of the wheel micro-tip are shown in Table 1.

Then, the diamond wheel micro-tip was used to grind a microgroove on the workpiece surface (see Figure 2b). A quartz glass substrate was chosen as workpiece. The diamond wheel micro-tip angle *α_v_* was equal to the microgroove angle *β_v_* [19]. The micro-grinding conditions are shown in Table 2.

## 5. Experiment and Measurement

**Figure 4:**
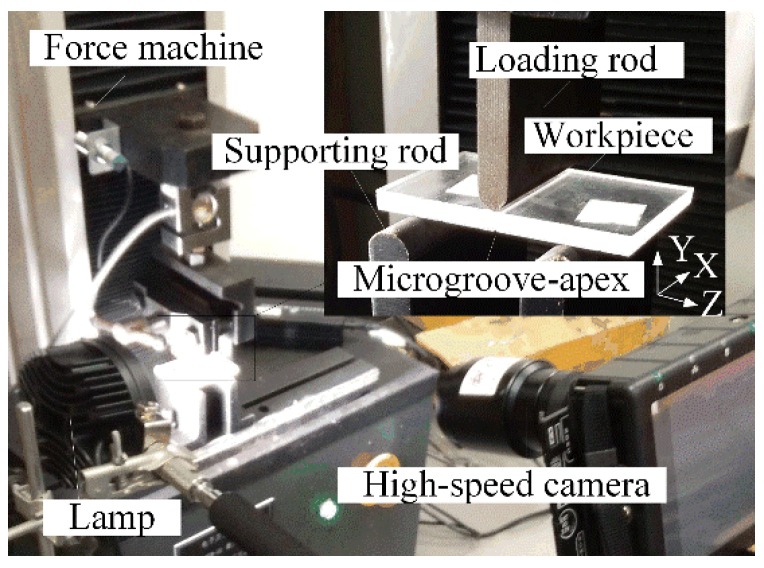
The experimental setup of micro-crack induced severing.

Figure 4 shows the experimental setup of micro-crack induced severing. It is based on its working principle (see Figure 1 and Figure 2). A WDW-05 electronic universal testing machine (Jinan Kason Testing Equipment Co., Ltd., Qingdao, Shandong, China) was employed to perform the loading and measure the on-line loading force *F*. A high-speed camera was used to record the propagation of micro-cracks during the loading process (see Figure 4). In the experiments, the microgroove angle *β_v_* was set as 60°, 90° and 120°, respectively. The loading rate *v* was set as 5 mm/min, 10 mm/min, 20 mm/min, 60 mm/min, 100 mm/min, 200 mm/min and 300 mm/min, respectively. In order to calculate ideal severing force F_c_^*^, the values of the auxiliary parameters such as *λ*_1_, *μ*_1_, *χ_b_*_1_, *χ_c_*_1_ and *χ_d_*_1_ in Equation (13) were given in Table 3. The mechanical properties of quartz glass were given in Table 4.

In order to compare the traditional scribing with the micro-crack induced severing, an SFT-QG500A glass-cutter machine (Shufeng technology co. Ltd., Shenzhen, China) was employed to perform the scribing-and-breaking severing experiments of quartz glass. The rolling scribing conditions were given by 120° in tungsten carbide wheel V-tip angle, 0.19 MPa in scribing pressure, 1 mm in setting depth and 30 mm/s in scribing speed.

## 6. Results and Discussions

### 6.1. Profile of Micro-Ground Microgroove on Workpiece Surface

**Figure 5:**
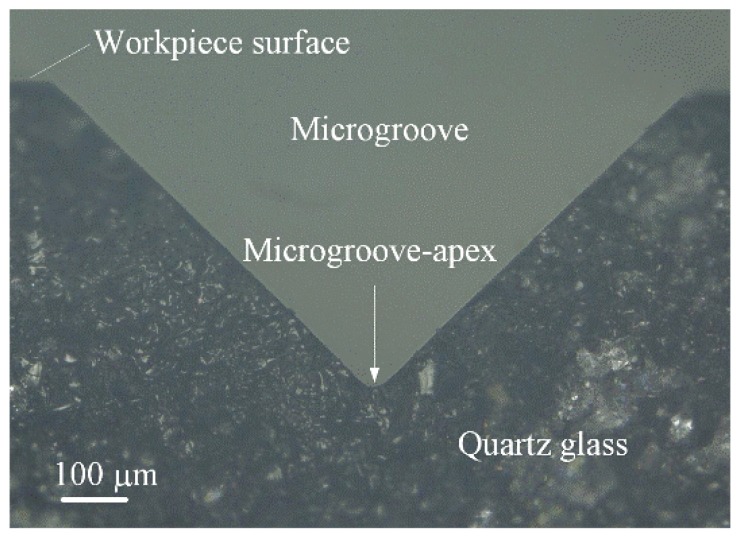
The microgroove profile of quartz glass after micro-grinding.

Figure 5 shows the microgroove profile of quartz glass after micro-grinding. It is shown that the microgroove was regular and its edges were smooth. No micro-cracks existed on the microgroove. This is because the grain cutting depths may be controlled to be less than the critical cutting depth transferred from brittle cutting to ductile cutting in micro-grinding, leading to a no-crack microgroove. According to the measured results of VHX-1000 microscope (Keyence, Osaka, Japan), the microgroove angle *β_v_* averagely reached 61.7°, 91.8° and 119.4° in contrast to the designed ones of 60°, 90° and 120°, respectively. The microgroove angle error was ±1.8°. Correspondingly, the microgroove heights *h_v_* were 545 μm, 495 μm and 479 μm, respectively. The microgroove-apex radius *r_v_* were 39.3 μm, 41.3 μm and 39.7 μm, respectively. The microgroove surface roughness *R_a_* was 80–100 nm. As a result, the micro-grinding was able to fabricate an accurate and smooth microgroove without any cracks around its apex. In contrast, the scribing produced the cracks along the scratch [2,3].

### 6.2. Severing Surface Topography

**Figure 6:**
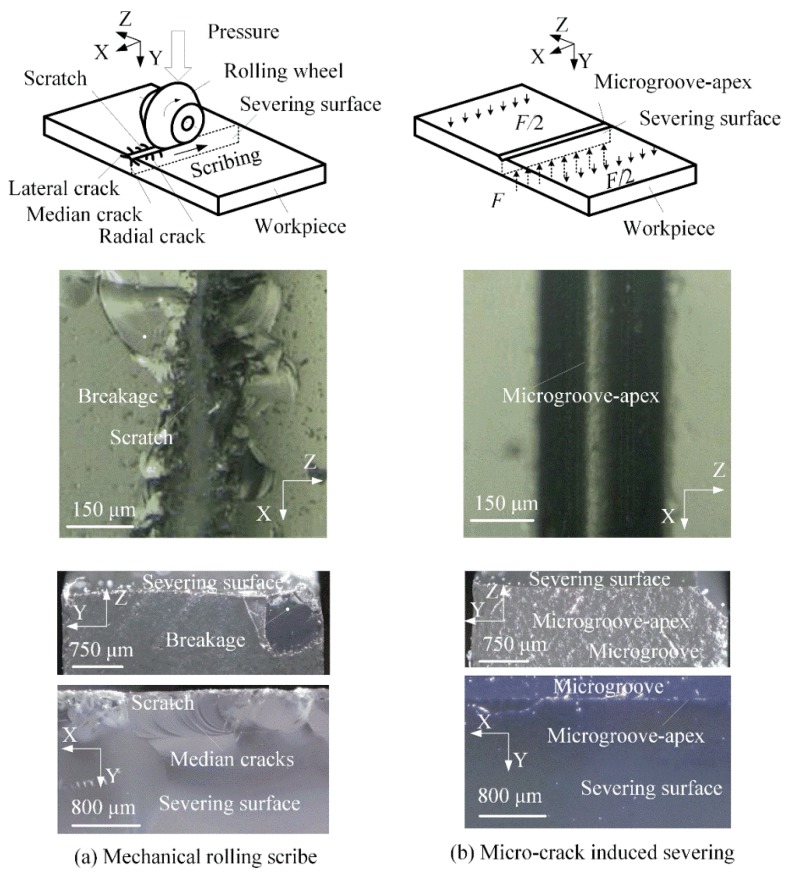
The severing surface topographies of quartz glass substrate: (**a**) mechanical rolling scribe (**b**) micro-crack induced severing.

Figure 6 shows the severing surface topographies of quartz glass in mechanical rolling scribing and micro-crack-induced severing. It is shown that breakages happened on the scratch microgroove edges in mechanical rolling scribing (see Figure 6a). This is because the pure mechanical compression produced the radial cracks, leading to edge cracks. It also produced median cracks, radial cracks and lateral cracks, leading to an uneven severing surface in break-severing. It was identical to the results in the scribing-and-breaking severing of ceramics and silica glass substrates [1,3]. In contrast, no breakages happened on the severing workpiece edges along the micro-ground microgroove-apex in micro-crack-induced severing (see Figure 6b). 

The severing surface edges were undamaged. The severing surface was flat and smooth. This is because the crack propagation was precisely induced along the micro-ground microgroove-apex in micro-crack-induced severing (see Figure 5). This also means that the accurate and smooth microgroove-apex without any micro-cracks was able to induce the accurate and smooth crack propagation in severing.

### 6.3. Loading Force and Loading Time versus Loading Rate

**Figure 7:**
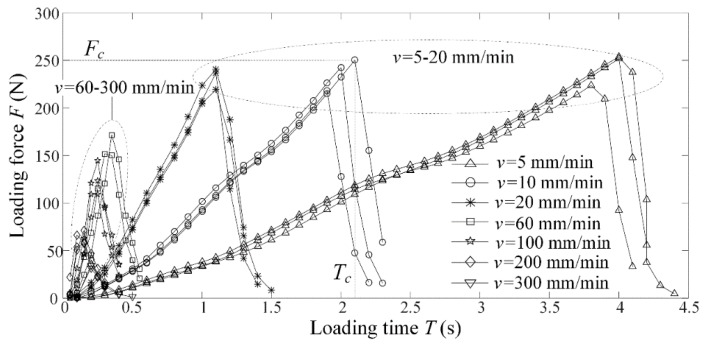
Loading force *F* and loading time *T* versus loading rate *v*.

Figure 7 shows the loading force *F* and loading time *T* versus loading rate *v*. Experimental results showed that the loading force *F* increased with increasing loading time *T* at beginning, but it rapidly decreased after the micro-crack propagation happened on the microgroove-apex. 

It was identical to the relationship between loading force and displacement in tensile fracture of notched polycrystalline graphite [23]. The critical loading force and time were regarded as severing force *F_c_* and severing time *T_c_*, respectively. In the case of loading rate *v* = 60–300 mm/min, the severing force rapidly increased with increasing loading time *T*, but it slowly increased in the case of *v* = 5–20 mm/min. This mean that there existed two different mechanisms in micro-crack induced severing. Their critical loading rate *v_c_* was distributed between 20 mm/min and 60 mm/min. As a result, the micro-crack induced severing may be distinguished by static severing (*v* < *v_c_*) and dynamic severing (*v* > *v_c_*), respectively.

### 6.4. Severing Force versus Microgroove-Apex Radius and Loading Rate

**Figure 8:**
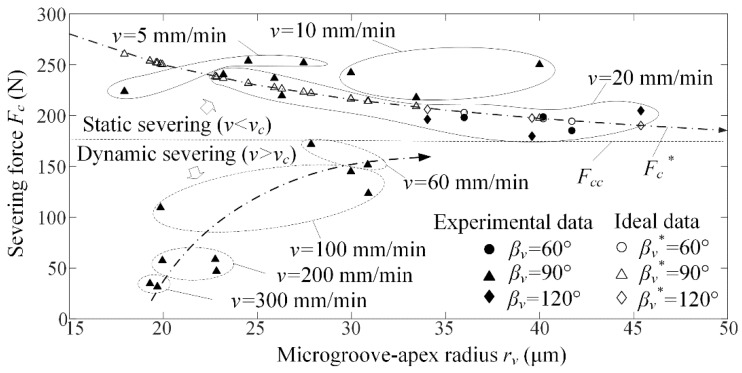
Severing force *F_c_* versus microgroove-apex radius *r_v_* for different loading rate *v* and microgroove angle *β_v_*.

Figure 8 shows the severing force *F_c_* versus microgroove-apex radius *r_v_* for different loading rate *v* and microgroove angle *β_v_*. Three experiments were accomplished for the same loading rate. The singularity was removed. It is shown that the severing force *F_c_* rapidly increased and slowly approach the critical severing force *F_cc_* with increasing microgroove-apex radius *r_v_* in dynamic severing, but it slowly decreased and gradually approach the critical severing force *F_cc_* in static severing. This mean that the static severing and dynamic severing produced different influence on severing force with reference to microgroove-apex radius. Moreover, the microgroove angle *β_v_* produced little influence on the severing force *F_c_*. The severing force *F_c_* averagely reached 226.5 N in static severing, but it averagely reached 116.2 N in dynamic severing. As a result, the static severing increased the severing force by 95% compared to the dynamic severing.

It is also seen that the severing force *F_c_* rapidly decreased with increasing loading rate *v* in dynamic severing. In contrast, it slowly decreased in static severing. However, the loading rate produced little influence on maximum loading force in low and high loading rate when the workpiece was thermoset epoxy resin [14]. When the microgroove-apex radius was larger than the critical value of 40–50 μm, the severing force was not dominated by the microgroove-apex radius and the loading rate. Moreover, the critical severing fore *F_cc_* ranged 160–180 N when the dynamic severing was transformed into static severing.

### 6.5. Prediction of Severing Force

In contrast to the ideal F_c_^*^, the experimental severing force *F_c_* was fitted to achieve *K_t_* as follows:

(14) $$K_{t}=-0.3741\beta_{v}+4.787$$

According to Equation (13), the ideal severing force F_c_^*^ may be described as follows:

(15) $$F_{c}^{*}=\frac{326.06{\left(0.4012r_{v}+0.0104\right)}^{0.4878}}{r_{v}^{0.4878}\left(1+0.5791{\left(1+\frac{0.0104}{0.4012r_{v}}\right)}^{-0.9179}\right)},\beta_{v}=60{}^{\circ}$$

(16) $$F_{c}^{*}=\frac{331.66{\left(0.3333r_{v}+0.0104\right)}^{0.4552}}{r_{v}^{0.4552}\left(1+0.54{\left(1+\frac{0.0104}{0.3333r_{v}}\right)}^{-0.8897}\right)},\beta_{v}=90{}^{\circ}$$

(17) $$F_{c}^{*}=\frac{313.83{\left(0.2481r_{v}+0.0104\right)}^{0.3843}}{r_{v}^{0.3843}\left(1+0.4319{\left(1+\frac{0.0104}{0.2481r_{v}}\right)}^{-0.8835}\right)},\beta_{v}=120{}^{\circ}$$

Using Equations (15)–(17), the results of ideal severing force F_c_^*^ were plotted in Figure 8. It is seen that the experimental cutting force was identical to the ideal severing force F_c_^*^ (see Figure 8). When the microgroove-apex radius was larger than the critical value of 40–50 μm. The ideal severing force F_c_^*^ was stabilized at 159.5–190.0 N, which was identical to the experimental results. This mean that the Equations (13)–(16) may be used to predict and control the severing force according to the microgroove height *h_v_*, angle *β_v_* and microgroove-apex radius *r_v_* in static severing.

### 6.6. Ideal Severing Force versus Microgroove Angle and Height

**Figure 9:**
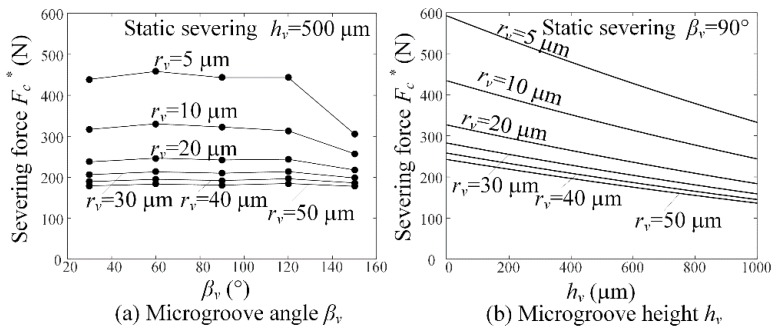
Ideal severing force F_c_^*^ versus microgroove parameters: (**a**) microgroove angle *β_v_* and (**b**) microgroove height *h_v_*.

Figure 9 shows the ideal severing force F_c_^*^ versus microgroove angle *β_v_* and height *h_v_* in the static severing. It is shown that the microgroove angle *β_v_* produced little influence on the severing force F_c_^*^ in the case of *β_v_* = 60–120°, but the severing force F_c_^*^ decreased with increasing microgroove *β_v_* in the case of *β_v_* > 120° and *h_v_* > 30 μm, respectively (see Figure 9a). It is also seen that the severing force F_c_^*^ decreased with increasing microgroove height *h_v_* and microgroove-apex radius *r_v_* (see in Figure 9b). When the microgroove-apex radius *r_v_* was larger than 40–50 μm, the severing force was not dominated by the microgroove angle and microgroove-apex radius.

### 6.7. Severing Time versus Microgroove-Apex Radius

**Figure 10:**
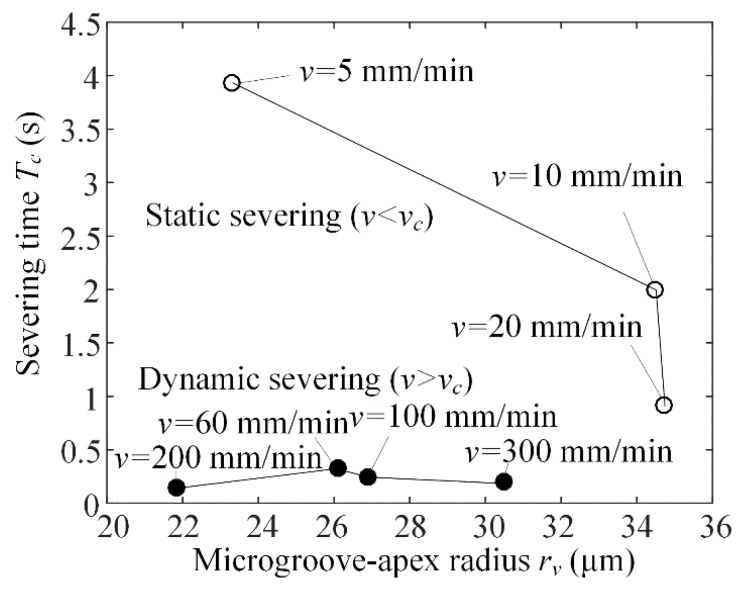
Severing time *T_c_* versus microgroove-apex radius *r_v_*.

Figure 10 shows the severing time *T_c_* versus microgroove-apex radius *r_v_*. The severing time *T_c_* was regarded as the mean value of experimental data at the same loading rate *v*. Experimental results showed that the severing time *T_c_* rapidly decreased with increasing microgroove-apex radius *r_v_* in static severing, but the microgroove-apex radius *r_v_* produced little influence on severing time in dynamic severing. Moreover, the severing time *T_c_* averagely reached 2.43 s in static severing, but it averagely reached 0.27 s in dynamic severing. Hence, the static severing increased the severing time by about 900% compared to the dynamic severing.

### 6.8. Severing form Errors versus Loading Rate

**Figure 11:**
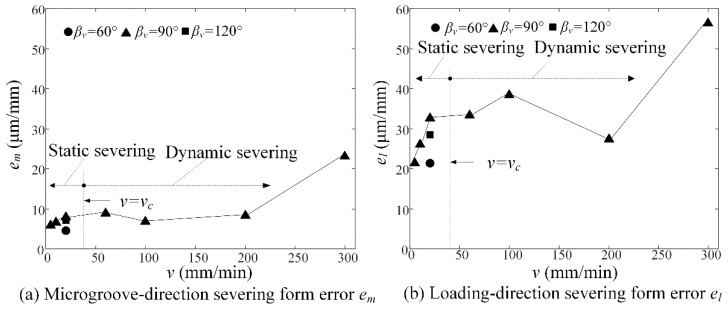
Severing form errors versus loading rate *v*: (**a**) microgroove-direction severing form error *e_m_* and (**b**) loading-direction severing form error *e_l_*.

Figure 11 shows the microgroove-direction *e_m_* and the loading-direction severing form errors *e_l_* versus loading rate *v* in micro-crack induced severing. Experimental results showed that the severing form errors gradually increased with increasing loading rate *v* in both static severing and dynamic severing. The microgroove-direction severing form error *e_m_* averagely reached 8.8 μm/mm (see Figure 11a), which was much less than the loading-direction severing form error of 31.7 μm/mm (see Figure 11b). This is because the microgroove direction was dominated by the accurate micro-ground microgroove-apex, but the loading direction depended on precision positioning and loading rate. Moreover, the static severing form error averagely reached 16.2 μm/mm, which was less than the dynamic severing form error of 25.3 μm/mm. The reason is that high loading rate easily produced the position deviation between workpiece and loading rod. Hence, the static severing may decrease the severing form error by 36% compared to the dynamic severing.

### 6.9. Severing Surface Roughness versus Loading Rate

**Figure 12:**
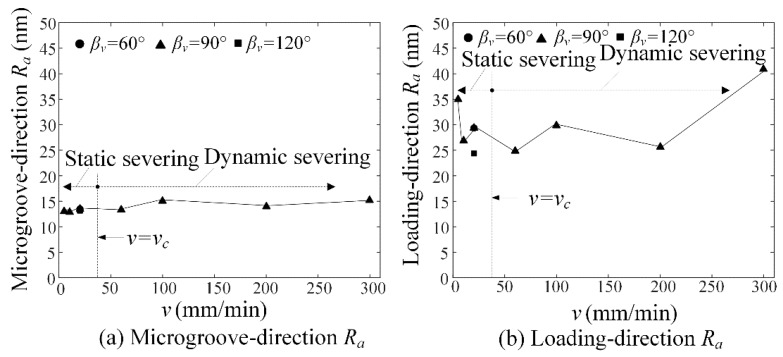
Severing surface roughness *R_a_* versus loading rate *v*: (**a**) microgroove-direction severing roughness and (**b**) loading-direction severing roughness.

Figure 12 shows the severing surface roughness *R_a_* versus loading rate *v*. Experimental results showed that the loading rate *v* had little influence on the severing surface roughness *R_a_* in both static severing and dynamic severing. However, the microgroove-direction severing surface roughness of 13.7 nm was much less than the loading-direction severing surface roughness of 29.6 nm. This mean that severing surface roughness could be dominated by material properties. Moreover, the static severing surface roughness of 19.69 nm was less than the dynamic severing surface roughness of 22.34 nm. Hence, the static severing surface roughness decreased by 12% compared to the dynamic severing. As a result, the micro-crack induced severing may produce smooth severing surface of quartz glass without any polishing.

### 6.10. Cracking Propagation Time and Loading Positions

**Figure 13:**
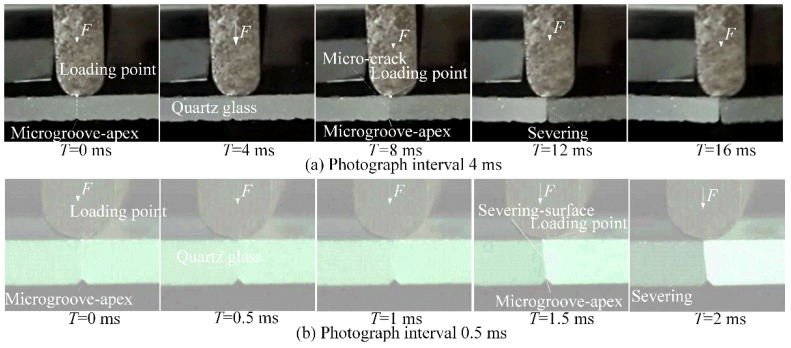
Micro-crack propagation process in static severing (*v* = 20 mm/min).

Figure 13 shows the micro-crack propagation process in static severing. It is shown that crack propagation occurred at the same time from the microgroove-apex and the loading point in 0.5 ms (see Figure 13a,b). This mean that the micro-crack was induced from the microgroove-apex to extend to the loading point. Hence, the loading positions dominated the severing form errors in micro-crack induced severing. This may explain why the severing form errors increased with increasing loading rate *v* (see Figure 11).

It is also found that the cracking propagation time reached 0.5 ms and less to achieve the smooth surface (see Figure 13b). In contrast, the severing time *T_c_* averagely reached 2430 ms in static severing (see Figure 10). This also mean that the severing time was much larger than the crack propagation time. Hence, the efficiency of micro-crack induced severing is dominated by the severing time rather than the crack propagation time.

## 7. Conclusions

1. Compared to a mechanical rolling scribe, the micro-crack-induced severing by a non-cracked microgroove-apex produces smooth severing edges without any breakages, median cracks, radial cracks and lateral cracks. The micro-grinding may machine the accurate and smooth microgroove-apex without any cracks.
2. In micro-crack-induced severing, the crack propagation occurred at the same time from the microgroove-apex and the loading point. The severing efficiency is dominated by the severing time rather than the crack propagation time. The severing energy and quality depend on the loading rate.
3. When the loading rate was less than 20–60 mm/min, the dynamic severing changes to static severing. In static severing, the severing force slowly decreases with increasing microgroove-apex radius, but it rapidly increases in dynamic severing. For the critical severing force of 160–180 N, the static severing force is about two times larger than the dynamic one.
4. The severing time rapidly decreases with increasing microgroove-apex radius in static severing, but it slowly increases in dynamic severing. It reaches on average 2.43 s and 0.27 s, respectively. The static severing increases the severing time by about 9 times compared to the dynamic severing. In contrast, the cracking propagation time reaches 0.5 ms and less.
5. The severing form error and the severing surface roughness reach 4.6–32.5 μm/mm and 12.8–34.9 nm in static severing and 6.9–56.3 μm/mm and 13.3–40.9 nm in dynamic severing, respectively. The static severing may decrease the severing form error by 36% and the severing surface roughness by 12% compared to the dynamic severing, respectively.
6. In static severing, the severing force may be modelled and predicted by microgroove-apex radius, microgroove angle and height. Theoretically, it decreases with increasing the microgroove height. In dynamic severing, it is little influenced by microgroove-apex radius and microgroove angle when the microgroove angle is less than 120°.

## Figures and Tables

**Table 1:** The truing conditions of diamond wheel micro-tip.

CNC grinder	SMART B818
Diamond wheel	SD600 (Metal-bond, Grain size: 24 μm), *D* = 150 mm, *w* = 4 mm
Dresser	#800 GC, Ceramic bond
Tool paths	V-shaped symmetrical, *α* = 60°, 90°, 120°
Truing parameters	*v_f_* = 500 mm/min, *N* = 2400 rpm, *a* = 20 μm, ∑*a* = 5 mm
Coolant	Water

**Table 2:** Micro-grinding conditions of microgroove on workpiece substrate.

CNC Grinder	SMART B818
Diamond wheel	SD600 (Metal-bond, grain size: 24 μm), *D* = 150 mm, *w* = 4 mm
Workpiece	Quartz glass
Grinding parameters	*v_f_* = 500 mm/min, *N* = 2400 rpm, *a* = 20 μm, ∑*a* = 500 μm,
Coolant	Water

**Table 3:** The auxiliary parameters.

*β_v_*	*λ*_1_	*μ*_1_	*χ*_*b*1_	*χ*_*c*1_	*χ*_*d*1_
60°	0.5122	−0.4057	1.3123	3.2832	0.0960
90°	0.5448	−0.3449	1.8414	2.5057	0.1046
120°	0.6157	−0.2678	3.0027	1.5150	0.0871

**Table 4:** Mechanical properties of quartz glass.

Property	Value
Young’s modulus [GPa]	77
Poisson’s ratio	0.17
Ultimate tensile strength *σ_u_* [MPa]	50
Fracture toughness *K*_IC_ [MPa·m^0.5^]	0.81

## References

[1] Tomei N., Murakami K., Hashimoto T., Kitaichi M., Hirano S., Fukunishi T. (2015). Development of a scribing wheel for cutting ceramic substrates and its wheel scribing and breaking technology. JSAT.

[2] Ono T., Tanaka K. (2001). Effect of scribe-wheel dimensions on the cutting of AMLCD glass substrate. J. Soc. Inf. Disp..

[3] Pan C.T., Hsieh C.C., Su C.Y., Liu Z.S. (2008). Study of cutting quality for TFT-LCD glass substrate. Int. J. Adv. Manuf. Technol..

[4] Hasegawa R., Matsusaka S., Hidai H., Chiba A., Morita N., Onuma T. (2016). In-process estimation of fracture surface morphology during wheel scribing of a glass sheet by high-speed photoelastic observation. Precis. Eng..

[5] Liao Y.S., Yang G.M., Hsu Y.S. (2010). Vibration assisted scribing process on LCD glass substrate. Int. J. Mach. Tools Manuf..

[6] Tsai C.H., Lin B.C. (2007). Laser cutting with controlled fracture and pre-bending applied to LCD glass separation. Int. J. Adv. Manuf. Technol..

[7] Tsai C.H., Huang B.W. (2008). Diamond scribing and laser breaking for LCD glass substrates. J. Mater. Process. Technol..

[8] Yamamoto K., Hasaka N., Morita H., Ohmura E. (2009). Crack propagation in glass by laser irradiation along laser scribed line. J. Manuf. Sci. Eng..

[9] Jiao J., Wang X. (2009). Cutting glass substrates with dual-laser beams. Opt. Lasers Eng..

[10] Kang M.G., Kim C., Lee Y.J., Kim S.Y., Lee H. (2016). Picosecond UV laser induced scribing of polyethylene terephthalate (PET) films for the enhancement of their flexibility. Opt. Laser Technol..

[11] Filippi S., Lazzarin P., Tovo R. (2002). Developments of some explicit formulas useful to describe elastic stress fields ahead of notches in plates. Int. J. Solids Struct..

[12] Ayatollahi M.R., Torabi A.R. (2010). Brittle fracture in rounded-tip V-shaped notches. Mater. Des..

[13] Torabi A.R., Firoozabadi M., Ayatollahi M.R. (2015). Brittle fracture analysis of blunt V-notches under compression. Int. J. Solids Struct..

[14] Ayatollahi M.R., Torabi A.R., Firoozabadi M. (2015). Theoretical and experimental investigation of brittle fracture in V-notched PMMA specimens under compressive loading. Eng. Fract. Mech..

[15] Barati E., Alizadeh Y. (2012). A notch root radius to attain minimum fracture loads in plates weakened by U-notches under Mode I loading. Sci. Iran..

[16] Kanchanomai C., Rattananon S., Soni M. (2005). Effects of loading rate on fracture behavior and mechanism of thermoset epoxy resin. Polym. Test..

[17] Hsieh C.C., Yao S.C. (2006). Evaporative heat transfer characteristics of a water spray on micro-structured silicon surfaces. Int. J. Heat Mass Transf..

[18] Dhupal D., Doloi B., Bhattacharyya B. (2008). Pulsed Nd: YAG laser turning of micro-groove on aluminum oxide ceramic (Al_2_O_3_). Int. J. Mach. Tools Manuf..

[19] Xie J., Zhuo Y.W., Tan T.W. (2011). Experimental study on fabrication and evaluation of micro pyramid-structured silicon surface using a V-tip of diamond grinding wheel. Precis. Eng..

[20] Ritchie R.O., Knott J.F., Rice J.R. (1973). On the relationship between critical tensile stress and fracture toughness in mild steel. J. Mech. Phys. Solids.

[21] Susmel L., Taylor D. (2008). The theory of critical distances to predict static strength of notched brittle components subjected to mixed-mode loading. Eng. Fract. Mech..

[22] Li L., Guo W.G., Yu X., Fu D.X. (2017). Mechanical behavior of ceramic-metal joint under quasi-static and dynamic four point bending: Microstructures damage and mechanisms. Ceram. Int..

[23] Ayatollahi M.R., Torabi A.R. (2010). Tensile fracture in notched polycrystalline graphite specimens. Carbon.

